# Crystal structure of post-perovskite-type CaIrO_3_ reinvestigated: new insights into atomic thermal vibration behaviors

**DOI:** 10.1107/S2056989015015649

**Published:** 2015-08-29

**Authors:** Akihiko Nakatsuka, Kazumasa Sugiyama, Akira Yoneda, Keiko Fujiwara, Akira Yoshiasa

**Affiliations:** a Graduate School of Science and Engineering, Yamaguchi University, Ube 755-8611, Japan; b Institute for Materials Research, Tohoku University, Sendai 980-8577, Japan; c Institute for Study of the Earth’s Interior, Okayama University, Misasa 682-0193, Japan; d Graduate School of Science and Technology, Kumamoto University, Kumamoto 860-8555, Japan

**Keywords:** crystal structure, redetermination, calcium iridium(IV) trioxide, post-perovskite, thermal vibration

## Abstract

Single crystals of the title compound, the post-perovskite-type CaIrO_3_ [calcium iridium(IV) trioxide], have been grown from a CaCl_2_ flux at atmospheric pressure. The crystal structure consists of an alternate stacking of IrO_6_ octa­hedral layers and CaO_8_ hendeca­hedral layers along [010]. Chains formed by edge-sharing of IrO_6_ octa­hedra (point-group symmetry 2/*m*..) run along [100] and are inter­connected along [001] by sharing apical O atoms to build up the IrO_6_ octa­hedral layers. Chains formed by face-sharing of CaO_8_ hendeca­hedra (point-group symmetry *m*2*m*) run along [100] and are inter­connected along [001] by edge-sharing to build up the CaO_8_ hendeca­hedral layers. The IrO_6_ octa­hedral layers and CaO_8_ hendeca­hedral layers are inter­connected by sharing edges. The present structure refinement using a high-power X-ray source confirms the atomic positions determined by Hirai *et al.* (2009 ▸) [*Z. Kristallogr.* **224**, 345–350], who had revised our previous report [Sugahara *et al.* (2008 ▸). *Am. Mineral.* **93**, 1148–1152]. However, the displacement ellipsoids of the Ir and Ca atoms based on the present refinement can be approximated as uniaxial ellipsoids elongating along [100], unlike those reported by Hirai *et al.* (2009 ▸). This suggests that the thermal vibrations of the Ir and Ca atoms are mutually suppressed towards the Ir⋯Ca direction across the shared edge because of the dominant repulsion between the two atoms.

## Abstract 2

Single crystals of CaIrO_3_ were grown from a CaCl_2_ flux at atmospheric pressure and crystallized with the post-perovskite type of structure. The crystal structure is reinvestigated on the basis of single-crystal X-ray diffraction data measured using a high-power X-ray source, and the atomic thermal vibration behavior is discussed in terms of the coordination environments.

## Chemical context   

The ortho­rhom­bic perovskite-type MgSiO_3_, the dominant constituent in the Earth’s lower mantle, is now believed to undergo the phase transition to the so-called ‘post-perovskite-type structure’, associated with the D′′ seismic discontinuity, at 125 GPa and 2500 K (Murakami *et al.*, 2004 ▸; Tsuchiya *et al.*, 2004 ▸; Oganov & Ono, 2004 ▸; Iitaka *et al.*, 2004 ▸; Mao *et al.*, 2004 ▸; Ono & Oganov, 2005 ▸; Wentzcovitch *et al.*, 2006 ▸; Shieh *et al.*, 2006 ▸). Since the discovery of the post-perovskite-type MgSiO_3_, several ortho­rhom­bic *A* ^2+^ *B* ^4+^O_3_ perovskite-type compounds have been found to transform into the post-perovskite-type structure under high pressure and high temperature (Kojitani *et al.*, 2007 ▸; Yamaura *et al.*, 2009 ▸; Tateno *et al.*, 2010 ▸). Meanwhile, CaIrO_3_ is known to be one of the few post-perovskite-type compounds stable at ambient conditions (Rodi & Babel, 1965 ▸; McDaniel & Schneider, 1972 ▸). The post-perovskite-type CaIrO_3_ has attracted much attention in the field of Earth science as an excellent low-pressure analogue of the post-perovskite-type MgSiO_3_ (see, for example, Niwa *et al.*, 2007 ▸; Tsuchiya & Tsuchiya, 2007 ▸; Yoneda *et al.*, 2014 ▸).

The crystal structure of the post-perovskite-type CaIrO_3_ was first proposed by Rodi & Babel (1965 ▸) on the basis of a single-crystal X-ray diffraction experiment, but incorrect atomic positions were reported. Recently, we have successfully grown single crystals of the post-perovskite-type CaIrO_3_ and refined the crystal structure of this compound on the basis of single-crystal X-ray diffraction data measured using a sealed X-ray tube (40 kV, 30 mA) as the radiation source (Sugahara *et al.*, 2008 ▸). However, the measured intensity data were rather weak and their accuracy was rather low, because thin needle-like crystals were obtained and the selected crystal for the intensity measurements had a poor grade of crystallinity. This resulted in rather large reliability indices [*R*(*F*) = 0.064, *wR*(*F*) = 0.065 for 377 reflections] and in structural parameters with rather large uncertainties. In particular, the resulting displace­ment ellipsoids were unusually elongated or flattened. Subsequently, Hirai *et al.* (2009 ▸) reinvestigated the crystal structure of the post-perovskite-type CaIrO_3_ by single-crystal X-ray diffraction and conducted structure refinements for two different crystals using two different types of diffractometers. The two independent refinements showed convergent results with much better reliability indices [*R*(*F* ^2^) = 0.013, *wR*(*F* ^2^) = 0.031 for 365 reflections; *R*(*F* ^2^) = 0.007, *wR*(*F* ^2^) = 0.008 for 149 reflections] and structural parameters with reasonably smaller uncertainties. Consequently, Hirai *et al.* (2009 ▸) concluded that the displacement ellipsoids had no significant anisotropies in contradiction to our previous report (Sugahara *et al.*, 2008 ▸), but provided no further details of the atomic thermal vibration behaviors. Their X-ray diffraction experiments were conducted under the operating conditions of 2θ_max_ = 80° at 45 kV/40 mA for one crystal and 2θ_max_ = 55° at 50 kV/85 mA for the other crystal. These operating conditions with a low X-ray power and a relatively low 2θ_max_ value may be insufficient for the determination of reliable atomic displacement parameters (ADPs).

In the present study, the crystal structure of the post-perovskite-type CaIrO_3_ was reinvestigated on the basis of single-crystal X-ray diffraction data measured over a much wider 2θ range using a high-power rotating-anode X-ray generator (60 kV, 250 mA). Special attention to exclude the influence of multiple scattering effects and secondary extinction effects on ADPs was paid as far as possible during data reduction and structure refinement procedures, as will be described in Section 5. On the basis of the resulting structural parameters, the validity of the crystal structure proposed by Hirai *et al.* (2009 ▸) is examined and the detailed atomic thermal vibration behaviors are discussed.

## Structural commentary   

**Figure 1:**
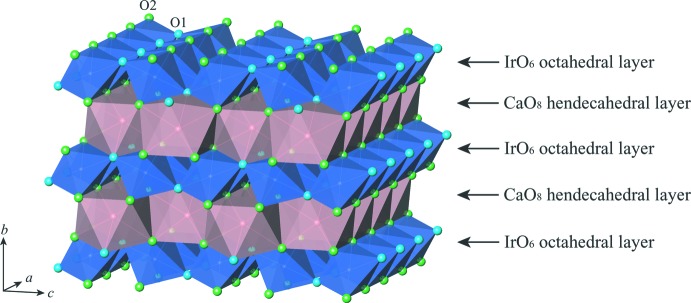
Polyhedral representation of the CaIrO_3_ post-perovskite-type structure, composed of the alternate stacking of IrO_6_ octa­hedral layers and CaO_8_ hendeca­hedral layers along [010].

**Figure 2:**
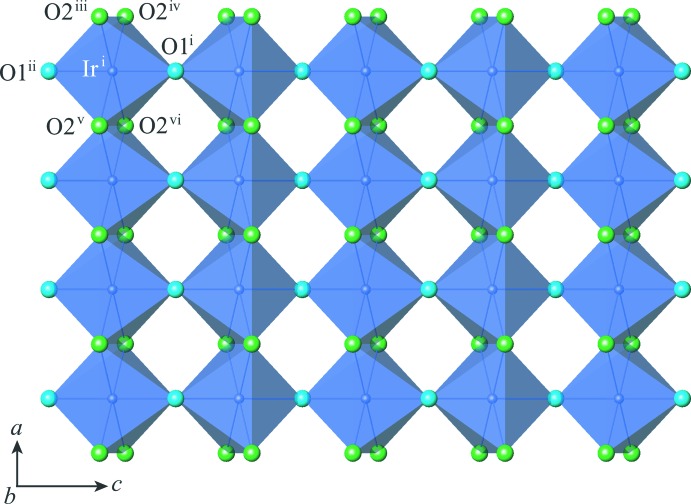
Polyhedral view of an IrO_6_ octa­hedral layer projected on (010). Symmetry codes: (i) *x* + 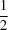, *y* + 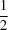, *z*; (ii) *x* + 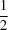, −*y* + 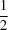, −*z*; (iii) *x* + 1, −*y* + 1, *z* − 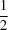; (iv) *x* + 1, *y*, −*z* + 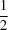; (v) *x*, −*y* + 1, *z* − 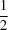; (vi) *x*, *y*, −*z* + 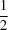.

**Figure 3:**
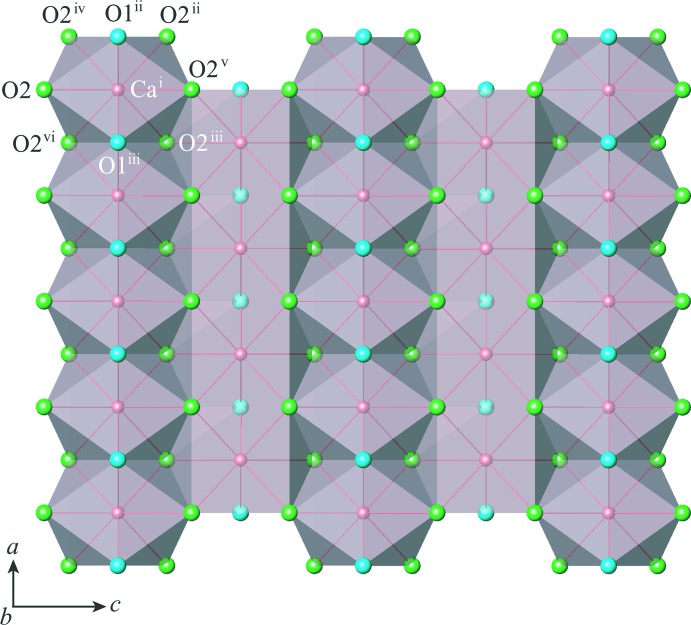
Polyhedral view of a CaO_8_ hendeca­hedral layer projected on (010). Symmetry codes: (i) *x*, −*y* + 1, −*z* + 1; (ii) *x* + 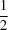, −*y* + 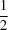, *z* + 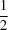; (iii) *x* − 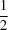, −*y* + 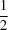, *z* + 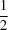; (iv) *x* + 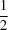, −*y* + 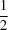, −*z* + 1; (v) *x*, *y*, −*z* + 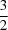; (vi) *x* − 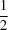, −*y* + 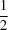, −*z* + 1.

The post-perovskite-type phase of CaIrO_3_ crystallizes in the space group *Cmcm*. The crystal structure consists of IrO_6_ octa­hedral layers and CaO_8_ hendeca­hedral layers stacked alternately along [010] (Fig. 1 ▸). The Ca and Ir atoms occupy Wyckoff positions 4*c* and 4*a*, respectively. The O atoms occupy two non-equivalent sites: O1 at Wyckoff position 4*c* and O2 at Wyckoff position 8*f*. The site symmetries are *m*2*m* for Ca, 2/*m*.. for Ir, *m*2*m* for O1 and *m*.. for O2. Ca—O and Ir—O bond lengths are listed in Table 1 ▸. In the IrO_6_ octa­hedral layers (Fig. 2 ▸), chains of IrO_6_ octa­hedra along [100] are formed by sharing O2⋯O2 edges, and these chains are inter­connected along [001] by sharing the apical O1 atoms. In the CaO_8_ hendeca­hedral layers (Fig. 3 ▸), chains of CaO_8_ hendeca­hedra along [100] are formed by sharing O2⋯O1⋯O2 faces, and these chains are inter­connected along [001] by sharing O2⋯O2 edges. The alternate stacking of IrO_6_ octa­hedral layers and CaO_8_ hendeca­hedral layers along [010] results from sharing O1⋯O2 and O2⋯O2 edges between both layers. Further details of the general description of the crystal structure are provided in our previous paper (Sugahara *et al.*, 2008 ▸).

## Atomic thermal vibration behaviors   

**Figure 4:**
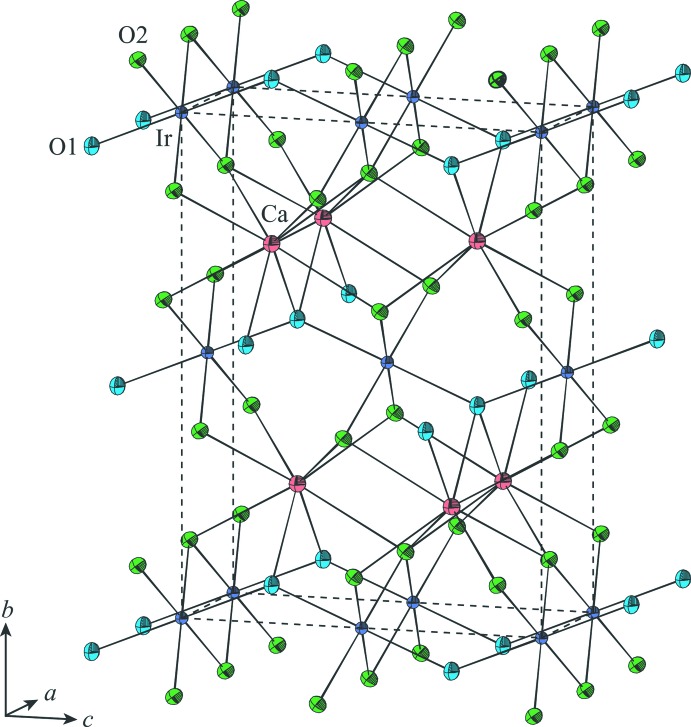
Unit cell of the CaIrO_3_ post-perovskite with displacement ellipsoids drawn at the 80% probability level.

In the present structure refinement using a high-power X-ray source, the accuracy of the refined structural parameters was considerably improved compared with our previous report (Sugahara *et al.*, 2008 ▸), being comparable to those reported by Hirai *et al.* (2009 ▸). The resulting positional parameters also show excellent consistency with those reported by Hirai *et al.* (2009 ▸). On the other hand, the present displacement ellipsoids (Fig. 4 ▸) are different from those given by Hirai *et al.* (2009 ▸). They considered that the thermal vibrations of the Ir and Ca atoms exhibited no significant anisotropies, but in fact the reported displacement ellipsoids of both atoms were somewhat elongated parallel to the (100) plane. In contrast, the mean-square displacements (MSDs) of both atoms obtained from the present refinement are as follows: Ir, 0.00316 (5) Å^2^ along the shortest ellipsoid axis, 0.00319 (5) Å^2^ along the inter­mediate one and 0.00387 (6) Å^2^ along the longest one; Ca, 0.0055 (3) Å^2^ along the shortest ellipsoid axis, 0.0058 (3) Å^2^ along the inter­mediate one and 0.0065 (3) Å^2^ along the longest one. Here, in both atoms, the longest ellipsoid axes are just in the [100] direction and the inter­mediate and the shortest ones are within the (100) plane. The present results indicate that the MSDs of both atoms are significantly the largest in the [100] direction, in contradiction to the report of Hirai *et al.* (2009 ▸), although the thermal vibrations of both atoms only exhibit small anisotropies.

As understood from the MSDs shown above, the displacement ellipsoid of the Ir atom is very close to a uniaxial ellipsoid elongating along [100]. The Ir⋯Ca direction across the O2⋯O2 shared edge between the IrO_6_ octa­hedron and CaO_8_ hendeca­hedron is parallel to the (100) plane; hence, this direction can be considered as the direction of nearly the smallest MSD of the Ir atom although it deviates by 10.1° from the direction of the shortest ellipsoid axis. The ellipsoid axes of the Ca atom are in the [100], [010] and [001] directions by requirements of its site symmetry, but its displacement ellipsoid can also be approximated as a uniaxial ellipsoid elongating along [100]. The Ir⋯Ca direction across the O2⋯O2 shared edge can thus be characterized also as the direction of nearly the smallest MSD of the Ca atom. These suggest that the dominant Ir⋯Ca repulsion across the O2⋯O2 shared edge suppresses the mutual thermal vibrations of both atoms towards the Ir⋯Ca direction. Indeed, the Ir⋯Ca distance [= 3.0678 (9) Å] is the shortest of the cation–cation distances [*cf.* 3.1466 (5) Å for the Ir⋯Ir distance across the O2⋯O2 shared edge between IrO_6_ octa­hedra; 3.1466 (5) Å for the Ca⋯Ca distance across the O2⋯O1⋯O2 shared face between CaO_8_ hendeca­hedra; 3.4501 (8) Å for the Ir⋯Ca distance across the O1⋯O2 shared edge between the IrO_6_ octa­hedron and CaO_8_ hendeca­hedron; 3.9755 (3) Å for the Ca⋯Ca distance across the O2⋯O2 shared edge between CaO_8_ hendeca­hedra].

## Synthesis and crystallization   

The reagents Ca(OH)_2_ and Ir were employed as the starting materials, and mixed together with CaCl_2_ in the molar ratio Ca(OH)_2_:Ir:CaCl_2_ = 1:1:10. The mixture was heated in air at 1100 K for 8 h, and then cooled gradually to 600 K at a rate of 10 K h^−1^. Dark reddish-brown crystals of the post-perovskite-type CaIrO_3_ with a thin needle shape were grown from the CaCl_2_ flux. The crystals were isolated by dissolving the solid­ified CaCl_2_ melt with distilled water.

## Refinement   

A total of 2593 intensity data up to 2θ_max_ = 100° were collected. After the intensity data were corrected for Lorentz-polarization factors and absorption effects (ψ-scan method; North *et al.*, 1968 ▸), they were averaged in Laue symmetry *mmm* to give 692 independent reflections. Of these, independent reflections with *F*_o_ ≤ 3σ(*F*_o_) were eliminated. Even if independent reflections had intensities of *F*_o_ > 3σ(*F*_o_) after averaging, those averaged from a data set of equivalent reflections including reflection(s) with *F*_o_ ≤ 3σ(*F*_o_) were also discarded since these reflections were potentially affected by multiple scattering. Moreover, independent reflections with (sin θ)/λ < 0.334 Å^−1^ were eliminated to reduce secondary extinction effects and to avoid dependence on atomic charge as far as possible in the choice of atomic scattering factors. Finally, 412 independent reflections were used in the present refinement. Several correction models for the secondary extinction effects were attempted during the refinement, and the isotropic correction of Type II (Becker & Coppens, 1974*a* ▸,*b* ▸) with a Gaussian particle-size-distribution model yielded the best fit. The reliability indices converged to *R*(*F*) = 0.0108 and *wR*(*F*) = 0.0104 for 412 reflections, comparable to those of Hirai *et al.* (2009 ▸), and were dramatically improved in comparison with those of our previous report (Sugahara *et al.*, 2008 ▸). Crystal data, data collection and structure refinement details are summarized in Table 2 ▸.

## Supplementary Material

Crystal structure: contains datablock(s) General, I. DOI: 10.1107/S2056989015015649/wm5181sup1.cif

Structure factors: contains datablock(s) I. DOI: 10.1107/S2056989015015649/wm5181Isup2.hkl

CCDC reference: 1419830

Additional supporting information:  crystallographic information; 3D view; checkCIF report

## Figures and Tables

**Table 1:** Selected bond lengths ()

CaO1^i^	2.333(3)	IrO1	1.9722(15)
CaO2^i^	2.460(2)	IrO2^iii^	2.0488(18)
CaO2^ii^	2.506(3)		

Symmetry codes: (i) 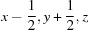; (ii) 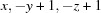; (iii) 
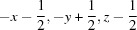
.

**Table 2:** Experimental details

Crystal data	
Chemical formula	CaIrO_3_
*M*_r_	280.30
Crystal system, space group	Orthorhombic, *C* *m* *c* *m*
Temperature (K)	298
*a*, *b*, *c* ()	3.1466(5), 9.8690(16), 7.3019(5)
*V* (^3^)	226.75(6)
*Z*	4
Radiation type	Mo *K*
(mm^1^)	61.02
Crystal size (mm)	0.20 0.01 0.01

Data collection	
Diffractometer	Rigaku AFC7R
Absorption correction	scan (North *et al.*, 1968 ▸)
*T*_min_, *T*_max_	0.486, 0.543
No. of measured, independent and observed [*F* > 3.0(*F*)] reflections	2593, 692, 438
*R*_int_	0.019
(sin /)_max_ (^1^)	1.078

Refinement	
*R*[*F* > 3(*F* ^2^)], *wR*(*F*), *S*	0.011, 0.010, 1.56
No. of reflections	412
No. of parameters	20
_max_, _min_ (e ^3^)	1.21, 1.89

Computer programs: *WinAFC* (Rigaku, 1999 ▸), *RADY* (Sasaki, 1987 ▸), *ATOMS for Windows* (Dowty, 2000 ▸) and *publCIF* (Westrip, 2010 ▸).

## supplementary crystallographic information

### Crystal data

**Table:**

CaIrO_3_	*F*(000) = 484
*M**_r_* = 280.30	*D*_x_ = 8.214 Mg m^−^^3^
Orthorhombic, *C**m**c**m*	Mo *K*α radiation, λ = 0.71069 Å
Hall symbol: -C 2c 2	Cell parameters from 25 reflections
*a* = 3.1466 (5) Å	θ = 22.5–25.0°
*b* = 9.8690 (16) Å	µ = 61.02 mm^−^^1^
*c* = 7.3019 (5) Å	*T* = 298 K
*V* = 226.75 (6) Å^3^	Needle, dark reddish-brown
*Z* = 4	0.20 × 0.01 × 0.01 mm

### Data collection

**Table:**

Rigaku AFC7R diffractometer	*R*_int_ = 0.019
ω–2θ scans	θ_max_ = 50.0°
Absorption correction: ψ scan (North *et al.*, 1968)	*h* = 0→6
*T*_min_ = 0.486, *T*_max_ = 0.543	*k* = −21→21
2593 measured reflections	*l* = −15→15
692 independent reflections	3 standard reflections every 150 reflections
438 reflections with *F* > 3.0σ(*F*)	intensity decay: none

### Refinement

**Table:**

Refinement on *F*	Weighting scheme based on measured s.u.'s *w* = 1/σ^2^(*F*)
*R*[*F*^2^ > 2σ(*F*^2^)] = 0.019	(Δ/σ)_max_ = 0.0003
*wR*(*F*^2^) = 0.021	Δρ_max_ = 1.21 e Å^−^^3^
*S* = 1.56	Δρ_min_ = −1.89 e Å^−^^3^
412 reflections	Extinction correction: isotropic Type II (Becker & Coppens, 1974*a,b*)
20 parameters	Extinction coefficient: 1.50E4 (5)

### Fractional atomic coordinates and isotropic or equivalent isotropic displacement parameters (Å^2^)

**Table:**

	*x*	*y*	*z*	*U*_iso_*/*U*_eq_	
Ca	0.0000	0.7502 (1)	0.2500	0.0060 (2)	
Ir	0.0000	0.0000	0.0000	0.00340 (5)	
O1	0.0000	0.0756 (4)	0.2500	0.0065 (11)	
O2	0.0000	0.3724 (3)	0.4495 (3)	0.0059 (7)	

### Atomic displacement parameters (Å^2^)

**Table:**

	*U*^11^	*U*^22^	*U*^33^	*U*^12^	*U*^13^	*U*^23^
Ca	0.0065 (3)	0.0055 (3)	0.0058 (3)	0.0000	0.0000	0.0000
Ir	0.00387 (6)	0.00317 (5)	0.00317 (5)	0.0000	0.0000	0.00001 (10)
O1	0.0084 (13)	0.0067 (12)	0.0045 (10)	0.0000	0.0000	0.0000
O2	0.0064 (9)	0.0054 (8)	0.0058 (7)	0.0000	0.0000	−0.0007 (6)

### Geometric parameters (Å, º)

**Table:**

Ca—O1^i^	2.333 (3)	Ir—Ir^xxv^	3.6510 (3)
Ca—O1^ii^	2.333 (3)	O1—Ir	1.9722 (15)
Ca—O2^i^	2.460 (2)	O1—Ir^xxv^	1.9722 (15)
Ca—O2^ii^	2.460 (2)	O1—Ca^xxiii^	2.333 (3)
Ca—O2^iii^	2.460 (2)	O1—Ca^xxiv^	2.333 (3)
Ca—O2^iv^	2.460 (2)	O1—O1^xii^	3.1466 (5)
Ca—O2^v^	2.506 (3)	O1—O1^xiii^	3.1466 (5)
Ca—O2^vi^	2.506 (3)	O1—O1^vii^	3.944 (3)
Ir—O1	1.9722 (15)	O1—O1^xxv^	3.944 (3)
Ir—O1^vii^	1.9722 (15)	O1—O2^viii^	2.748 (2)
Ir—O2^viii^	2.0488 (18)	O1—O2^ix^	2.748 (2)
Ir—O2^ix^	2.0488 (18)	O1—O2^xxvi^	2.748 (2)
Ir—O2^x^	2.0488 (18)	O1—O2^xxvii^	2.748 (2)
Ir—O2^xi^	2.0488 (18)	O1—O2^xxiii^	2.936 (3)
Ca—Ca^xii^	3.1466 (5)	O1—O2^xxiv^	2.936 (3)
Ca—Ca^xiii^	3.1466 (5)	O1—O2^x^	2.936 (3)
Ca—Ca^xiv^	3.9755 (3)	O1—O2^xi^	2.936 (3)
Ca—Ca^xv^	3.9755 (3)	O1—O2^xxviii^	3.271 (4)
Ca—Ca^xvi^	3.9755 (3)	O1—O2	3.271 (4)
Ca—Ca^xvii^	3.9755 (3)	O2—Ir^xx^	2.0488 (18)
Ca—Ir^xviii^	3.0678 (9)	O2—Ir^xxi^	2.0488 (18)
Ca—Ir^xix^	3.0678 (9)	O2—Ca^xxiii^	2.460 (2)
Ca—Ir^i^	3.4501 (8)	O2—Ca^xxiv^	2.460 (2)
Ca—Ir^ii^	3.4501 (8)	O2—Ca^xix^	2.506 (3)
Ca—Ir^xx^	3.4501 (8)	O2—O1^xx^	2.748 (2)
Ca—Ir^xxi^	3.4501 (8)	O2—O1^xxi^	2.748 (2)
Ir—Ca^xxii^	3.0678 (9)	O2—O1^i^	2.936 (3)
Ir—Ca^vi^	3.0678 (9)	O2—O1^ii^	2.936 (3)
Ir—Ca^xxiii^	3.4501 (8)	O2—O1	3.271 (4)
Ir—Ca^xxiv^	3.4501 (8)	O2—O2^v^	2.625 (5)
Ir—Ca^viii^	3.4501 (8)	O2—O2^xxviii^	2.913 (5)
Ir—Ca^ix^	3.4501 (8)	O2—O2^xxvi^	2.976 (5)
Ir—Ir^xii^	3.1466 (5)	O2—O2^xxvii^	2.976 (5)
Ir—Ir^xiii^	3.1466 (5)	O2—O2^xii^	3.1466 (5)
Ir—Ir^vii^	3.6510 (3)	O2—O2^xiii^	3.1466 (5)
			
O1^i^—Ca—O1^ii^	84.82 (13)	O2^vi^—Ca—O2^iii^	73.63 (9)
O1^i^—Ca—O2^i^	86.02 (7)	O2^vi^—Ca—O2^iv^	73.63 (9)
O1^i^—Ca—O2^ii^	142.50 (6)	O2^vi^—Ca—O2^v^	122.28 (13)
O1^i^—Ca—O2^vi^	69.12 (5)	O2^iii^—Ca—O2^iv^	79.52 (8)
O1^i^—Ca—O2^iii^	86.02 (7)	O2^iii^—Ca—O2^v^	139.04 (5)
O1^i^—Ca—O2^iv^	142.50 (6)	O2^iv^—Ca—O2^v^	139.04 (5)
O1^i^—Ca—O2^v^	69.12 (5)	O1—Ir—O1^vii^	180.00
O1^ii^—Ca—O2^i^	142.50 (6)	O1—Ir—O2^viii^	86.22 (10)
O1^ii^—Ca—O2^ii^	86.02 (7)	O1—Ir—O2^ix^	86.22 (10)
O1^ii^—Ca—O2^vi^	69.12 (5)	O1—Ir—O2^x^	93.78 (10)
O1^ii^—Ca—O2^iii^	142.50 (6)	O1—Ir—O2^xi^	93.78 (10)
O1^ii^—Ca—O2^iv^	86.02 (7)	O1^vii^—Ir—O2^viii^	93.78 (10)
O1^ii^—Ca—O2^v^	69.12 (5)	O1^vii^—Ir—O2^ix^	93.78 (10)
O2^i^—Ca—O2^ii^	79.52 (8)	O1^vii^—Ir—O2^x^	86.22 (10)
O2^i^—Ca—O2^vi^	139.04 (5)	O1^vii^—Ir—O2^xi^	86.22 (10)
O2^i^—Ca—O2^iii^	72.61 (10)	O2^viii^—Ir—O2^ix^	100.33 (12)
O2^i^—Ca—O2^iv^	121.28 (13)	O2^viii^—Ir—O2^x^	79.67 (12)
O2^i^—Ca—O2^v^	73.63 (9)	O2^viii^—Ir—O2^xi^	179.97
O2^ii^—Ca—O2^vi^	139.04 (5)	O2^ix^—Ir—O2^x^	179.97
O2^ii^—Ca—O2^iii^	121.28 (13)	O2^ix^—Ir—O2^xi^	79.67 (12)
O2^ii^—Ca—O2^iv^	72.61 (10)	O2^x^—Ir—O2^xi^	100.33 (12)
O2^ii^—Ca—O2^v^	73.63 (9)		

Symmetry codes: (i) *x*−1/2, *y*+1/2, *z*; (ii) *x*+1/2, *y*+1/2, *z*; (iii) −*x*−1/2, *y*+1/2, −*z*+1/2; (iv) −*x*+1/2, *y*+1/2, −*z*+1/2; (v) *x*, −*y*+1, −*z*+1; (vi) −*x*, −*y*+1, *z*−1/2; (vii) −*x*, −*y*, *z*−1/2; (viii) −*x*−1/2, −*y*+1/2, *z*−1/2; (ix) −*x*+1/2, −*y*+1/2, *z*−1/2; (x) −*x*−1/2, *y*−1/2, −*z*+1/2; (xi) −*x*+1/2, *y*−1/2, −*z*+1/2; (xii) *x*−1, *y*, *z*; (xiii) *x*+1, *y*, *z*; (xiv) −*x*−1/2, −*y*+3/2, *z*−1/2; (xv) −*x*−1/2, −*y*+3/2, *z*+1/2; (xvi) −*x*+1/2, −*y*+3/2, *z*−1/2; (xvii) −*x*+1/2, −*y*+3/2, *z*+1/2; (xviii) *x*, *y*+1, *z*; (xix) −*x*, −*y*+1, *z*+1/2; (xx) −*x*−1/2, −*y*+1/2, *z*+1/2; (xxi) −*x*+1/2, −*y*+1/2, *z*+1/2; (xxii) *x*, *y*−1, *z*; (xxiii) *x*−1/2, *y*−1/2, *z*; (xxiv) *x*+1/2, *y*−1/2, *z*; (xxv) −*x*, −*y*, *z*+1/2; (xxvi) *x*−1/2, −*y*+1/2, −*z*+1; (xxvii) *x*+1/2, −*y*+1/2, −*z*+1; (xxviii) −*x*, *y*, −*z*+1/2.
